# Exploring Micro-Eukaryotic Diversity in the Gut: Co-occurrence of *Blastocystis* Subtypes and Other Protists in Zoo Animals

**DOI:** 10.3389/fmicb.2020.00288

**Published:** 2020-02-25

**Authors:** Emma L. Betts, Eleni Gentekaki, Anastasios D. Tsaousis

**Affiliations:** ^1^Laboratory of Molecular and Evolutionary Parasitology, RAPID Group, School of Biosciences, University of Kent, Canterbury, United Kingdom; ^2^School of Science, Mae Fah Luang University, Chiang Rai, Thailand; ^3^Gut Microbiome Research Group, Mae Fah Luang University, Chiang Rai, Thailand

**Keywords:** *Blastocystis*, genetic diversity, subtyping, co-occurrence, phylogeny, micro-eukaryome

## Abstract

*Blastocystis* is a genetically diverse microbial eukaryote thriving in the gut of humans and other animals. While *Blastocystis* has been linked with gastrointestinal disorders, its pathogenicity remains controversial. Previous reports have suggested that one out of six humans could be carrying *Blastocystis* in their gut, while the numbers could be even higher in animals. Most studies on *Blastocystis* are either exclusively targeting the organism itself and/or the associated prokaryotic microbiome, while co-occurrence of other microbial eukaryotes has been mainly ignored. Herein, we aimed to explore presence and genetic diversity of *Blastocystis* along with the commonly occurring eukaryotes *Cryptosporidium*, *Eimeria*, *Entamoeba* and *Giardia* in the gut of asymptomatic animals from two conservation parks in the United Kingdom. Building upon a previous study, a total of 231 fecal samples were collected from 38 vertebrates, which included 12 carnivorous and 26 non-carnivorous species. None of the animals examined herein showed gastrointestinal symptoms. The barcoding region of the small subunit ribosomal RNA was used for subtyping of *Blastocystis*. Overall, 47% of animal species were positive for *Blastocystis*. Twenty six percent of samples carried more than one subtypes, including the newly identified hosts Scottish wildcat, bongo and lynx. Fifty three percent of samples carried at least another microbial eukaryote. Herewith, we discuss potential implications of these findings and the increasingly blurred definition of microbial parasites.

## Introduction

The gut microbiome comprises the collective genomes of microbial symbionts and is composed of bacteria, fungi, viruses and protists within the gastrointestinal (GI) tract of a host (Blaser, 2014). Though literature associated with bacterial microbiota is increasing, studies on the rest of the microbiome components are just beginning to surface. Historically, presence of protists in the gut has been considered as parasitism, thus these microbial eukaryotes have been subject to rigorous elimination in both humans and other animals (Parfrey et al., 2011). Despite this, current data demonstrates that some protists are more common than previously thought, raising the possibility of commensalistic or even mutualistic roles in the gut ecosystem (Lukes et al., 2015; Chudnovskiy et al., 2016). In this regard, no other protist has been studied more extensively than the anaerobic stramenopile *Blastocystis*. Its prevalence in humans has been estimated to a staggering one billion (Stensvold, 2012). Though a similar estimation for animals is not available, data from numerous animal studies covering broad range of hosts strongly suggest that colonization rate in animals is likely higher than in humans.

*Blastocystis* is extremely heterogeneous genetically (Gentekaki et al., 2017). Based on the SSU rRNA gene, *Blastocystis* from avian and mammalian hosts is divided into 17 subtypes, which are considered separate species (Stensvold and Clark, 2016b). Nonetheless, there are many sequences originating from ectothermic hosts that do not belong to any of the designated subtypes (Yoshikawa et al., 2016). The various subtypes of *Blastocystis* do not seem to be host-specific. For example, ST1 to ST9 have been identified in humans, but also in other hosts (Stensvold and Clark, 2016b). The exception seems to be ST9, which has yet to be identified in a non-human host (Stensvold and Clark, 2016a). ST10 to ST17 have been found only in animals so far, with the exception of ST12, which has also been identified in humans (Ramirez et al., 2016).

Though *Blastocystis* has been found in individuals with gastrointestinal symptoms, asymptomatic carriage is also common (Scanlan et al., 2014; AbuOdeh et al., 2016; Nieves-Ramirez et al., 2018; Yowang et al., 2018; Mardani Kataki et al., 2019). *In vitro* experiments using cell lines have shown the invasion potential of some strains/subtypes of *Blastocystis* (Puthia et al., 2008; Wawrzyniak et al., 2012), with no evidence to date that this also occurs *in vivo* (Clark et al., 2013). Experimental infections in mouse models have been achieved only after an inoculum of considerable size (up to 4 × 10^7^) is administered (Moe et al., 1997; Elwakil and Hewedi, 2010). Recent studies on animals have shown that *Blastocystis* exists asymptomatically in a broad array of hosts (Betts et al., 2018; Wang et al., 2018b). Collectively, these findings highlight the uncertainty surrounding pathogenicity status of *Blastocystis* in both humans and other animals.

Presence of multiple *Blastocystis* subtypes in humans is not often reported (Whipps et al., 2010; Scanlan and Stensvold, 2013). To our knowledge, only a few reports have demonstrated mixed colonization in animals (AbuOdeh et al., 2016; Cian et al., 2017; Betts et al., 2018). In our previous work, Betts et al. (2018) examined *Blastocystis* distribution in a wildlife park in the United Kingdom, and identified various genetic isolates in a number of different animals across the park. Importantly, we also demonstrated presence of up to four subtypes in healthy captive animals (Betts et al., 2018). At that time, while microscopically screening the fecal samples, we noted presence of other protists as well. Most previous studies have been focused on identifying single target protist species, but only a few have focused on co-occurrence of multiple microbial eukaryotes in the gut. Herein, we have expanded the study area to include an additional wildlife park. We aimed to further characterize presence of *Blastocystis* isolates along with additional microbial eukaryotes across a broad range of taxa in the two parks.

## Materials and Methods

### Study Sites

Two zoos situated in the Southeast, United Kingdom were sampled in this study: 1) Wildwood Conservation Park, Herne Bay, Kent, United Kingdom (51°19′54.1″N 1°07′10.1″E). This is a small conservation park housing native vertebrate and invertebrate species from the United Kingdom and mainland Europe with the exception of the red-necked wallaby (*Macropus rufogriseus*). The park is actively involved in breeding and re-introduction programs for native animals including the European water vole (*Arvicola amphibious*) and Scottish wildcat (*Felis silvestris silvestris*), and 2) Howletts Wild Animal park, Canterbury, Kent, United Kingdom (51°16′11.8″N 1°09′25.0″E). This is a large zoo with over 400 animals from 50 vertebrate and invertebrate species from across the globe. The zoo has a large primate collection, including one of the largest family groups of western lowland gorilla (*Gorilla gorilla gorilla*) in the world. The zoo is involved in a number of re-introductory schemes, mainly into national parks. Both zoos closely monitor animal health, through licensed veterinarians once a month. To our knowledge none of the animals in this study presented symptomatic gastrointestinal diseases or diarrhea.

### Sample Collection

A total of 231 fresh fecal samples have been collected from 38 vertebrate species between July 2016 and March 2019 (Supplementary Table S1). One hundred and eighteen samples were from a previous collection (Accession numbers of *Blastocystis* positive samples: MF186640-MF186709; Betts et al., 2018) and the rest were newly collected. Sixty-seven of these samples were from nine vertebrate species collected from Howletts Zoo between November 2017 and February 2019 and the remaining samples were collected form 31 species at Wildwood. Samples from gray wolf (*Canis lupus*) and European bison (*Bison bonasus*) were collected from both zoos. Sampling covered a total of 33 mammalian species, four bird species and one reptile (Supplementary Table S1). In both zoos, a minimum of one fecal sample was collected from each enclosure. In enclosures where more than one animal resided, between two and five samples were collected, each of which was considered as individual sample. For some water voles (*Arvicola amphibious*), a number of repeat collections were carried out over the course of 12 months (Supplementary Table S1). Fresh fecal samples were collected in the morning either before or shortly after enclosures were cleaned. For some animals, including avian species and the reptile; where age of fecal sample is difficult to determine, multiple samples were collected. Zookeepers supervised all collections.

Once collected, fecal samples were stored at 4°C in sterile falcon tubes within 1 h of collection until DNA extraction. In some instances, heat fixed slides were prepared. Within an hour of sampling, a small amount of fecal sample from the water voles and other randomly selected animals were separately inoculated in four sterile falcon tubes containing the following media: two tubes containing modified LYSGM [16⋅07 mM potassium phosphate dibasic, 2⋅94 mM potassium phosphate monobasic, 128⋅34 mM sodium chloride, 2⋅5 g L^–1^ yeast extract, 0⋅5 g L^–1^ liver extract, 5% adult bovine (Sigma)/horse serum (Gibco); modified TYSGM-9, without mucin (Diamond, 1982)^1^ ], two tubes of TYM (22⋅2 g L^–1^ trypticase peptone, 11⋅1 g L^–1^ yeast extract, 16⋅23 mM maltose, 9⋅17 mML-cysteine, 1⋅26 mM L-ascorbic acid, 5⋅1 mM potassium phosphate dibasic, 6⋅53 mM potassium phosphate monobasic) (Diamond, 1957, 1983) enriched with 5% fetal bovine serum (FBS; Sigma) and 2 tubes with 0⋅5% Liver Digest (LD) medium (0⋅5 g L^–1^ Oxoid liver extract). The tubes were incubated at 35°C. samples were examined for *Blastocystis* under the microscope every 3–5 days. After initially leaving the cultures for 2 weeks, they were subcultured every 10 days.

### DNA Extraction, Amplification of Target Gene and Molecular Characterization

Genomic DNA was extracted directly from a minimum of 250 mg of fresh fecal sample or culture pellet using the Microbiome DNA Purification Kit Purelink (Fisher, United Kingdom) to the manufacturer’s instructions. DNA was eluted in 100 μl elution buffer and aliquotted. The working stock was stored at −20°C, while the rest was placed at −80°C for long-term storage. Extracted DNA was used for the polymerase chain reaction (PCR) with specific primers targeting regions of interest (Supplementary Table S2). PCR was carried out using the 2X PCRBIO Taq DNA Polymerase (PCRBIOSYSTEMS). Reagents per 25 μl reaction were as follows: PCRBIO Taq mix, 0.4 μM forward primer, 0.4 μM reverse primer, 19 μl nuclease free water and 2 μl DNA (ranging in concentration 10–50 ng/μl). Details of amplification conditions for all species in this study are provided in Table 1.

**TABLE 1:** Summary of amplification conditions from this study.

Target Organism	Primer Pair	Primer Type	Initial Denaturation Conditions		Denaturation Conditions		Annealing Conditions		Extension Conditions		Cycle Number	Final Extension Conditions	
								
			Temp °C	Time min/s	Temp °C	Time min/s	Temp °C	Time min/s	Temp °C	Time min/s		Temp °C	Time min/s
*Blastocystis*	RD3/RD5	External	95	5 min	95	30 s	55	30 s	72	1 min 40 s	35	72	5 min
*Blastocystis*	RD5F/BhRDr	Internal	95	5 min	95	30 s	55	30 s	72	1 min 40 s	35	72	5 min
*Cryptosporidium*	CRY F1/CRY R1	External	94	2 min	94	50 s	53	50 s	72	1 min	24	72	10 min
*Cryptosporidium*	CRY F2/CRY R2	Internal	94	2 min	94	50	56	30 s	72	1 min	30	72	10 min
*Giardia*	RH11/RH4	-	96	2 min	96	45 s	58	30 s	72	45 s	30	72	4 min
*Eimeria*	EIF1/EIR3	External	94	5 min	94	30 s	57	30 s	72	2 mins	30	72	10 min
*Eimeria*	EIF3/EIR3	Internal	94	3 min	94	30 s	60	30 s	72	1 min 30 s	40	72	7 min
*Entamoeba*	542/543	-	94	5 min	94	30 s	55	30 s	72	30 s	35	72	2 min

Fragments amplified to the correct size were excised and extracted using the Thermo Scientific GeneJET Gel Extraction Kit (following manufacturer’s instructions) purified gel extracts were eluted in 30–50 μl of elution buffer. If PCR reactions were left for 7 days before ligation, a polyadenylation reaction was carried out with the following protocol: per reaction 0.25 μl GoTaq DNA Polymerase (Promega), 7 μl Gel extraction, 2 μl 5X GoTaq Buffer (Promega), 0.5 mM MgCl2, 2.5 mM dATP (Promega) and 0.3 μl nuclease water at 72°C for 30 min. 1.5 μl of polyadenylation product or gel extract was cloned using the pGEM-T easy vector system I (Promega) following manufacturer’s protocol. Between 3 and 10 colonies per transformation were grown in 5 ml overnight cultures. Plasmid DNA was extracted using the GeneJET Plasmid Miniprep Kit (following manufacturer’s instructions). Before sequencing, a restriction digest using *Eco*RI (Promega) was carried out to confirm fragment insertion, per 10 μl reaction, 0.25 μl *Eco*RI, 5 μl miniprep elution, 1 μl 10X buffer H and 3.75 μl dH20 was incubated at 37°C for 2 h and visualized on a 1.5% agarose gel. Positive samples were sequenced using both the T7 or SP6 universal primers by Eurofins, United Kingdom.

### Phylogenetic Analysis

Raw reads were trimmed to remove remaining vector fragments and unambiguous bases at the ends of the reads. BLAST search using the newly obtained sequences against the non-redundant (nr) database was used to identify sequence positive clones. A dataset was assembled including all new sequences in addition to reference sequences encompassing the breadth of diversity of *Blastocystis* and an alignment was carried out using MAFFT v.7 (Katoh and Toh, 2010). Alignment contained four outgroup taxa for a total of 171 taxa. After aligning with MAFFT, ambiguous positions were masked with trimAl (Capella-Gutierrez et al., 2009). Following trimming, the alignment contained 1326 positions. A maximum likelihood tree was constructed using the RAxML software version 8 (Stamatakis, 2014, 2015) on the online platform CIPRES (Miller et al., 2010).^2^ For each dataset bootstrap support was calculated from 1000 replicates.

## Results

### Culturing

*Blastocystis* was cultured in tubes containing both types of media. Isolates from fox, lynx, wallaby, elk and otter grew at 35°C, while the ones from water voles grew at room temperature (Supplementary Figure S1). We were unable to establish cultures from other hosts.

### Screening of Fecal Samples

Building upon sampling from a previous study, a total of 231 fecal samples from 38 vertebrate species were examined. It should be noted that the percent positive percentages herein are the minimum since PCR amplification rather than qPCR was used. *Blastocystis* was detected in 18/38 species (47%). A total of 255 clones were sequence positive for *Blastocystis*; 184 of these clones were from the current study. Of the 12-carnivorous species only three (pine marten, lynx and Scottish wild cat) were sequence positive for *Blastocystis* (25%, Table 2). There were no sequence positives for badger, European brown bear, otter, polecat, red and arctic foxes, stoat, gray and Iberian wolves, despite having multiple samples from different time points from these species. For non-carnivorous species, 15/26 (58%) were sequence positive, while barnacle and pink footed geese, four lined snake, hedgehog, water shrew, raven, red billed chough, black and brown rats, pied tamarind and black rhinoceros were negative (Table 2). *Blastocystis* was found in all artiodactyl species examined, but not all fecal samples were sequence positive. Sequence positive results for samples were as follows: Carnivora 3/50 (6%); Artiodactyla 20/36 (56%); Anseriformes 0/2 (0%); Squamata 0/1 (0%); Eulopotyphia 0/0 (0%); Passeriformes 0/4 (0%); Rodentia 29/81 (36%); Diprotodontia 2/5 (40%); Primates 27/43 (63%); Perissodactyla 0/0 (0%).

**TABLE 2:** Prevalence of *Blastocystis*, *Giardia*, *Cryptosporidium*, *Entamoeba*, and *Eimeria* in study animals.

Host	Scientific Name	Location	No. faecal samples collected	*Blastocystis* No. positive (% Positive)	*Giardia* No. Positive (% Positive)	*Cryptosporidium* No. Positive (% Positive)	*Entamoeba* No. Positive (% Positive)	*Eimeria* No. Positive (% Positive)
**Carnivora (*T* = 50)**								
Badger	*Meles meles*	Wildwood	2	0 (0)	0 (0)	0 (0)	0 (0)	1 (50)
European Brown Bear	*Ursus arctos arctos*	Wildwood	4	0 (0)	0 (0)	0 (0)	0 (0)	0 (0)
Lynx	*Lynx lynx*	Wildwood	5	2 (40)	0 (0)	0 (0)	0 (0)	0 (0)
Otter	*Lutra lutra*	Wildwood	7	0 (0)	0 (0)	0 (0)	0 (0)	0 (0)
Pine Marten	*Martes martes*	Wildwood	2	1 (50)	0 (0)	0 (0)	0 (0)	0 (0)
Polecat	*Mustela putarius*	Wildwood	1	0 (0)	0 (0)	0 (0)	0 (0)	0 (0)
Red Fox	*Vulpes vulpes*	Wildwood	3	0 (0)	0 (0)	0 (0)	0 (0)	0 (0)
Arctic Fox	*Vulpes lagopus*	Wildwood	2	0 (0)	0 (0)	0 (0)	0 (0)	0 (0)
Scottish Wild Cat	*Felis silvestris*	Wildwood	13	1 (8)	0 (0)	0 (0)	0 (0)	0 (0)
Stoat	*Mustela ermine*	Wildwood	3	0 (0)	0 (0)	0 (0)	0 (0)	0 (0)
Gray Wolf	*Canis lupus*	Howletts	3	0 (0)	0 (0)	0 (0)	0 (0)	1 (33)
Gray Wolf	*Canis lupus*	Wildwood	2	0 (0)	0 (0)	1 (50)	0 (0)	0 (0)
Iberian Wolf	*Canis lupus signatus*	Howletts	3	0 (0)	0 (0)	0 (0)	0 (0)	1 (33)
**Anseriformes (*T* = 2)**								
Barnacle Goose	*Branta leucopsis*	Wildwood	1	0 (0)	0 (0)	0 (0)	0 (0)	0 (0)
Pink Footed Goose	*Anser brachyrhynchus*	Wildwood	1	0 (0)	0 (0)	0 (0)	0 (0)	1 (100)
**Artiodactyla (*T* = 36)**								
Muntjac	*Muntiacus reevesi*	Wildwood	1	1 (100)	0 (0)	0 (0)	0 (0)	0 (0)
European Bison	*Bison bonasus*	Wildwood	5	3 (60)	0 (0)	0 (0)	2 (40)	1 (20)
European Bison	*Bison bonasus*	Howletts	4	0 (0)	0 (0)	0 (0)	2 (50)	2 (50)
Eurasian Elk	*Alces alces*	Wildwood	3	1 (33)	0 (0)	0 (0)	0 (0)	0 (0)
Pygmy Goat	*Capra aegagrus hircus*	Wildwood	2	2 (100)	0 (0)	0 (0)	2 (100)	0 (0)
Red Deer	*Cervus elaphus*	Wildwood	3	1 (33)	0 (0)	0 (0)	1 (33)	0 (0)
Reindeer	*Rangifer tarandus*	Wildwood	1	1 (100)	0 (0)	0 (0)	1 (100)	1 (100)
Soay Sheep	*Ovis aries*	Wildwood	1	1 (100)	0 (0)	0 (0)	1 (100)	0 (0)
Wild Boar	*Sus scrofa*	Wildwood	4	2 (50)	0 (0)	0 (0)	1 (25)	0 (0)
Red River Hog	*Potamochoerus porcus*	Howletts	6	3 (50)	0 (0)	1 (17)	1 (17)	0 (0)
Bongo	*Tragelaphus eurycerus*	Howletts	6	1 (17)	0 (0)	0 (0)	2 (33)	1 (17)
**Squamata (*T* = 1)**								
Four-lined Snake	*Elaphe quatuorlineata*	Wildwood	1	0 (0)	0 (0)	0 (0)	0 (0)	0 (0)
Eulopotyphla (*T* = 7)								
Hedgehog	*Erinaceus quatuorlineata*	Wildwood	1	0 (0)	0 (0)	0 (0)	0 (0)	0 (0)
Water Shrew	*Neomys fodiens*	Wildwood	6	0 (0)	0 (0)	0 (0)	1 (17)	0 (0)
**Passeriformes (*T* = 4)**								
Raven	*Corvus corax*	Wildwood	3	0 (0)	0 (0)	0 (0)	0 (0)	0 (0)
Red Billed Chough	*Pyrrhocorax pyrrhocorax*	Wildwood	1	0 (0)	0 (0)	0 (0)	0 (0)	0 (0)
**Rodentia (*T* = 81)**								
Black Rat	*Rattus rattus*	Wildwood	1	0 (0)	0 (0)	0 (0)	0 (0)	0 (0)
Brown Rat	*Rattus norvegicus*	Wildwood	1	0 (0)	0 (0)	0 (0)	0 (0)	0 (0)
Red Squirrel	*Sciurus vulgaris*	Wildwood	5	3 (60)	0 (0)	0 (0)	0 (0)	0 (0)
Water Vole	*Arvicola amphibious*	Wildwood	22	10 (45)	4 (18)	15 (68)	0 (0)	0 (0)
Water Vole	*Arvicola amphibious*	Tilbury	17	5 (29)	7 (41)	2 (12)	1 (6)	1 (6)
Water Vole	*Arvicola amphibious*	Bulphan	35	12 (34)	17 (49)	4 (11)	2 (6)	4 (11)
**Diprotodontia (*T* = 5)**								
Wallaby	*Macropus rufogriseus*	Wildwood	5	2 (40)	0 (0)	0 (0)	0 (0)	0 (0)
**Primates (*T* = 43)**								
Western Lowland Gorilla	*Gorilla gorilla gorilla*	Howletts	25	16 (64)	0 (0)	1 (4)	2 (8)	2 (8)
Javan Gibbon	*Hylobates moloch*	Howletts	13	11 (85)	1 (8)	6 (46)	1 (8)	0 (0)
Pied Tamarin	*Saguinus bicolor*	Howletts	5	0 (0)	0 (0)	0 (0)	0 (0)	0 (0)
**Perissodactyla (*T* = 2)**								
Black Rhinoceros	*Diceros bicornis*	Howletts	2	0 (0)	0 (0)	0 (0)	0 (0)	0 (0)

Regarding subtypes from cultures, we only looked at water voles as their cultures were numerous. We found only ST1 and ST4, while the rest of the STs found in the faces were not recovered.

### Diversity and Distribution of Subtypes

In total, 10 known subtypes were detected: ST1, ST2, ST3, ST4, ST5, ST8, ST10, ST13, ST14, and ST15 (Table 3). Of those, ST2, ST3, ST8 and ST15 were not found in our previous collection. Subtype 4 was the most commonly isolated, found in 83/255 (33%) clones across 11 species. This was followed by ST2, isolated from 80/255 samples (31%); ST10 27/255 (11%); ST1 26/255 (10%); ST14 17/255 (7%); ST5 13/255 (5%); ST3 and ST15 4/255 (2%); ST13 1/255 (0.4%). Three sequences grouped with the *B. lapemi* clade.

**TABLE 3:** *Blastocystis* subtypes and co-occurrence with other microbial eukaryotes.

Host	Location	No. sequence positive clones	*Blastocystis* ST											Co-occurrence with other protists
				
			ST1	ST2	ST3	ST4	ST5	ST8	ST10	ST13	ST14	ST15	ST?	
**Carnivora**														
Pine Marten	Wildwood	1	–	–	–	1/1	–	–	–	–	–	–		–
Lynx	Wildwood	2	–	1/2	–	–	–	–	–	–	1/2	–		–
Scottish Wild Cat	Wildwood	2	–	–	–	1/2	–	–	–	–	1/2			–
**Artiodactyla**														
Muntjac	Wildwood	1	–	–	–	–	–	–	–	1/1	–	–		–
European Bison	Wildwood	11	–	–	–	–	–	–	11/11		–	–		*Entamoeba, Eimeria*
Eurasian Elk	Wildwood	6	–	–	–	1/6	–	–	1/6	–	4/6	–		–
Pygmy Goat	Wildwood	3	1/3	–	–	–	–	–	1/3	–	1/3	–		*Entamoeba*
Red Deer	Wildwood	8	–	–	–	3/8	–	–	5/8	–	–	–		*Entamoeba*
Reindeer	Wildwood	1	–	–	–	–	–	–	1/1	–	–	–		*Entamoeba, Eimeria*
Soay Sheep	Wildwood	1	–	–	–	–	–	–	–	–	1/1	–		*Entamoeba*
Wild Boar	Wildwood	2	–	–	–	–	2/2	–	–	–	–	–		*Entamoeba*
Red River Hog	Howletts	5	–	–	–	–	5/5	–	–	–	–	–		*Cryptosporidium, Entamoeba*
Bongo	Howletts	10	–	–	–	–	–	–	5/10	–	5/10	–		*Entamoeba, Eimeria*
**Rodentia**														
Red Squirrel	Wildwood	4	–	3/4	–	1/4	–	–	–	–	–	–		–
Water Vole	Wildwood	30	3/30	–	–	24/30	–		1/30		–	–	2/30	*Cryptosporidium, Giardia*
Water Vole	Tilbury	28	–	–	–	25/28	–	–	–	–	–	3/28		*Cryptosporidium, Entamoeba, Giardia, Eimeria*
Water Vole	Bulphan	29	–	–	–	29/29	–	–	–	–	–	–		*Cryptosporidium, Entamoeba, Giardia, Eimeria*
**Diprotodontia**														
Wallaby	Wildwood	2	–	–	–	–	–	–	2/2	–	–	–		–
**Primates**														
Western Lowland Gorilla	Howletts	64	9/64	45/64	8/64	–	2/64	–	–	–	–	–		*Cryptosporidium, Entamoeba, Eimeria*
Javan Gibbon	Howletts	45	18/45	17/45	4/45	–	4/45	1/45	–	–	–	1/45		*Cryptosporidium, Giardia, Entamoeba*

All artiodactyls, except for the European Bison (*Bison bonasus*) housed at Howletts, had at least one positive ST identification. The subtypes found in this group coincided with published data with most isolates belonging to ST5, ST10 and ST14. ST5 was present in 6/36 (17%) samples; ST10 in 10/36 (28%) samples; ST14 in 7/36 (19%); ST4 in 2/36 (6%) samples; ST1 and ST13 both 1/36 (3%). 5/36 samples exhibited co-occurrence with two or more STs. The bongo calf (*Tragelaphus eurycerus*) –shared the same STs (10 and 14) with its mother as opposed to the father, who is housed separately and in whom we only detected ST14.

Eighty-one samples from four species belonging to the order Rodentia are presented in this study. Brown rat (*Rattus norvegicus*) and black rat (*Rattus rattus*) yielded no *Blastocystis* positive isolates. ST2 and ST4 were detected in three samples were from Red squirrel (*Sciurus vulgaris*). Water vole (*Arvicola amphibious*) samples accounted for a total of 26/81 (32%) positive *Blastocystis* samples and 88 positive clones. a total of 74 water vole samples have been taken to date, 26/74 (35%) are sequence positive for one or more STs. The large sample number is due to the sizable cohort in the study, which included repeat sampling over an extended period of time. Three groups of water vole were sampled: captive voles from Wildwood (22 samples) and wild caught voles from two areas in Essex, United Kingdom; Tilbury (17 samples) and Bulphan (35 samples). The wild caught voles were routinely screened over the course of 10–12 months. Amongst sequence positive samples the captive voles had a total of 30 positive clones obtained from 9/22 (41%) positive samples; Tilbury voles had 28 positive clones from 5/17 (29%) samples, while Bulphan voles yielded 29 clones from 11/35 (31%) positive samples. ST4 was the most commonly identified across both captive and wild voles, representing 76/88 (86%) of the clones and 23/26 (88%) samples. ST1, ST15 and a subtype placing with *B. lapemi* were all identified in two samples, ST1 and *B. lapemi* clade ST were isolated in captive voles, whereas ST15 was found in one wild vole across repeat sample time points. ST10 and ST14 were identified in one sample each from captive voles. Co-occurrence of two or more STs was identified in four voles, all of which were captive. ST4 was present in all of these co-occurrence instances along with ST1, ST10, ST14, and *B. lapemi* clade ST.

A total of 43 non-human primate (NHP) samples were collected from Howletts zoo as follows: 25 gorillas (*Gorilla gorilla gorilla*) samples from four family groups ranging in size (G1, G3 G4 and G5) and one individual were collected across two collection times, 13 Javan gibbon (*Hylobates moloch*) samples from individuals across seven groups ranging in size (A-G) and five pied tamarin (*Saguinus bicolor*) samples from group enclosures. Of these samples, 16/25 gorillas (64%); 11/13 (85%) Javan gibbons were sequence positive for at least one ST, while no *Blastocystis* was detected in any of the pied tamarins (Table 4). In terms of clones, for the gorillas, 64 positive clones were sequenced, of which 45/64 (70%) were ST2; 9/64 (14%) ST1; 8/64 (13%) ST3; and 2/64 (3%) were ST5. There were no notable differences observed among family groups. Specifically, all family groups had a relatively high incidence of ST2, while ST5 was only reported from family group 5. Co-colonization with two STs was seen in four of the gorilla samples (Table 4). The Javan gibbons represent one of the highest proportions of sequence positive clones for *Blastocystis* STs, from the 11 positive samples, 45 clones were sequenced. ST1 represented 18/45 (40%) of these clones; ST2 17/45 (38%); ST3 and ST5 both 4/45 (9%); ST8 1/45 (2%); ST15 1/45 (2%). Of the gibbon groups, Group F was the only one to not have any sequence positive data across two sample collections. Of all the groups, Group G was only sampled from once as its members were released to the wild between collections. Differences were observed among groups between the sample collections. For example, ST5 and ST15 were detected in Group B upon first collection, yet in the second ST1 and ST2 were found.

**TABLE 4:** *Blastocystis* subtyping in captive Javan gibbons (*Hylobates moloch*) and West Lowland gorillas (*Gorilla gorilla gorilla*) from two sample collections with co-occurrence of other protists within sampled groups.

Host	Collection Number	Family Group	No. Positive Sequences	*Blastocystis* ST						Co-occurrence with other protists
					
				ST1	ST2	ST3	ST5	ST8	ST15	
Javan Gibbon A	1	A	6	3			2	1		*Cryptosporidium*
Javan Gibbon B	1	B	3				2		1	–
Javan Gibbon C	1	C	3	2	1					–
Javan Gibbon D	1	D	3		3					*Giardia*
Javan Gibbon E	1	E	3		3					–
Javan Gibbon F	1	F	0							–
Javan Gibbon G	1	G	3		3					*Cryptosporidium*
Javan Gibbon A	2	A	4	1		3				–
Javan Gibbon B	2	B	2	1	1					*Cryptosporidium, Entamoeba*
Javan Gibbon C	2	C	12	11	1					–
Javan Gibbon D	2	D	2			2				–
Javan Gibbon E	2	E	4		4					–
Javan Gibbon F	2	F	0							–
Javan Gibbon G	2	G	0							N/A

**Host**	**Collection Number**	**Family Group**	**No. Positive Sequences**	***Blastocystis* ST**						**Co-occurrence with other protists**
							
				**ST1**	**ST2**	**ST3**	**ST5**			

West Lowland Gorilla 1	1	5	4		4					*Entamoeba*
West Lowland Gorilla 2	1	5	5		5					*Eimeria*
West Lowland Gorilla 3	1	4	6		6					–
West Lowland Gorilla 4	1	4	5		5					–
West Lowland Gorilla 5	1	3	4			4				–
West Lowland Gorilla 6	1	3	6		6					–
West Lowland Gorilla 7	1	3	6		5	1				–
West Lowland Gorilla 8	1	3	4		4					–
West Lowland Gorilla 9	1	3	1	1						–
West Lowland Gorilla 10	1	3	3	3						–
West Lowland Gorilla 1	2	1	2	2						–
West Lowland Gorilla 8	2	3	5		4	1				*Cryptosporidium*
West Lowland Gorilla 4	2	3	4		2	2				*Eimeria*
West Lowland Gorilla 10	2	4	3		3					–
West Lowland Gorilla 11	2	5	3	3						–
West Lowland Gorilla 12	2	5	3		1		2			–

In general, differences in ST distribution and prevalence are seen between the two zoos, the most obvious attribute to this is the differences in sampled taxa. Samples from Wildwood were comprised largely of members from the orders Rodentia, Artiodactyla and Carnivora, with Water voles and Scottish wild cats being sampled several times. Samples from Howletts were mainly from NHPs and other members of the Artiodactyla. The European bison and gray wolf were the only species sampled across both parks. Notably, *Blastocystis* was not isolated from any wolf or bison samples from Howletts, even though the bison housed at Wildwood and Howletts are related. The differences in ST distribution among the parks reflect the taxa housed within. Wildwood comprises largely of ST4 and ST10, STs commonly associated with rodents and hooved animals, whereas ST2, ST1 and ST5 are isolated on Howletts and are commonly associated with NHPs.

In total, 25 of the *Blastocystis* positive samples harbored more than one subtype; specifically, two subtypes were detected in 22 samples, three subtypes in two samples, while one sample contained four subtypes.

Newly generated sequences have been submitted to GenBank (MN526748- MN526930).

### Co-occurrence of *Blastocystis* and Other Protists

Fecal samples were screened for *Cryptosporidium*, *Eimeria*, *Entamoeba*, *Giardia* and *Isospora*. Of the 81 *Blastocystis* positive samples, 43 (53%) harbored at least one of the above-mentioned protists in addition to *Blastocystis* (Table 5). Of those, 35 samples had one additional protist as follows: 14 cases from samples of Rodentia (all water voles), 13 from Artiodactyla (three from European bisons, three from bongos, two from Red river hogs, two from pygmy goats, one from wild boar, one from soay sheep, and one from red deer) and eight from NHPs (four from gorillas and three from Javan gibbons). Seven samples carried *Blastocystis* and two other protists: four Rodentia (all from water voles), two NHPs (both from Javan gibbons), and one from Artiodactyla (reindeer). A single sample from water vole was found with three other protists. The widest range of host species where co-occurrence was noted in the Artiodactyla. *Cryptosporidium* was detected in 31 (13%) samples and co-occurred with *Blastocystis* in 19 cases (61%); 22 (9%) samples were positive *Entamoeba*, 14 of which (64%) were found with *Blastocystis*; 29 (12%) samples harbored *Giardia* which co-occurred with *Blastocystis* in 10 cases (35%); 17 (7%) samples were positive for *Eimeria*, while nine were found with *Blastocystis*. Of the three (1%) *Isospora* positive samples, none co-occurred with *Blastocystis*.

**TABLE 5:** Co-occurrence of *Blastocystis* with other microbial eukaryotes.

Sample	Order	Location	*Blastocystis* ST	*Cryptosporidium*	*Giardia*	*Eimeria*	*Entamoeba*	*Isospora*
Water Vole TB30.1	Rodentia	Tilbury	4	yes	yes	yes		
Javan Gibbon Group D	Primate	Howletts	2	yes	yes			
Water Vole R22	Rodentia	Wildwood	4	yes	yes			
Water Vole TB32.1	Rodentia	Tilbury	4	yes	yes			
Reindeer	Artiodactyla	Wildwood	10			yes	yes	
Water Vole TB29.1	Rodentia	Tilbury	15			yes	yes	
Javan Gibbon Group B	Primate	Howletts	1, 2	yes			yes	
Water Vole Q52	Rodentia	Wildwood	unknown	yes	yes			
Javan Gibbon Group G	Primate	Howletts	2	yes				
Western Lowland Gorilla 1 G5	Primate	Howletts	2				yes	
Western Lowland Gorilla 2 G5	Primate	Howletts	2			yes		
Water Vole C3	Rodentia	Wildwood	4	yes				
Water Vole C3	Rodentia	Wildwood	4	yes				
Water Vole C4	Rodentia	Wildwood	4	yes				
Water Vole C4	Rodentia	Wildwood	4	yes				
Water Vole PP01.2	Rodentia	Bulphan	4	yes				
Water Vole PP03.1	Rodentia	Bulphan	4		yes			
Water Vole PP03.2	Rodentia	Bulphan	4		yes			
Water Vole PP03.3	Rodentia	Bulphan	4		yes			
Water Vole PP03.4	Rodentia	Bulphan	4		yes			
Water Vole PP04.1	Rodentia	Bulphan	4		yes			
Water Vole PP05.2	Rodentia	Bulphan	4			yes		
Water Vole PP05.3	Rodentia	Bulphan	4			yes		
Red River Hog 2	Artiodactyla	Howletts	5	yes				
Red River Hog 3	Artiodactyla	Howletts	5				yes	
Wild Boar 1	Artiodactyla	Wildwood	5				yes	
European Bison 1	Artiodactyla	Wildwood	10				yes	
European Bison 1	Artiodactyla	Wildwood	10			yes		
European Bison 2	Artiodactyla	Wildwood	10				yes	
Bongo M	Artiodactyla	Howletts	14				yes	
Pygmy Goat 1	Artiodactyla	Wildwood	14				yes	
Soay Sheep	Artiodactyla	Wildwood	14				yes	
Water Vole TB29.2	Rodentia	Tilbury	15		yes			
Pygmy Goat 2	Artiodactyla	Wildwood	1, 10				yes	
Javan Gibbon Group C	Primate	Howletts	1, 2	yes				
Water Vole R12	Rodentia	Wildwood	1, 4	yes				
Javan Gibbon Group A	Primate	Howletts	1, 5, 8	yes				
Bongo Calf	Artiodactyla	Howletts	10 14				yes	
Bongo F	Artiodactyla	Howletts	10, 14			yes		
Western Lowland Gorilla 8 G3	Primate	Howletts	2, 3	yes				
Western Lowland Gorilla 4 G3	Primate	Howletts	2,3			yes		
Red Deer 1	Artiodactyla	Wildwood	4, 10				yes	
Water Vole Q99	Rodentia	Wildwood	4, unknown	yes				
Javan Gibbon Group B	Primate	Howletts	5, 15	yes				

### Phylogenetic Analysis

**FIGURE 1:**
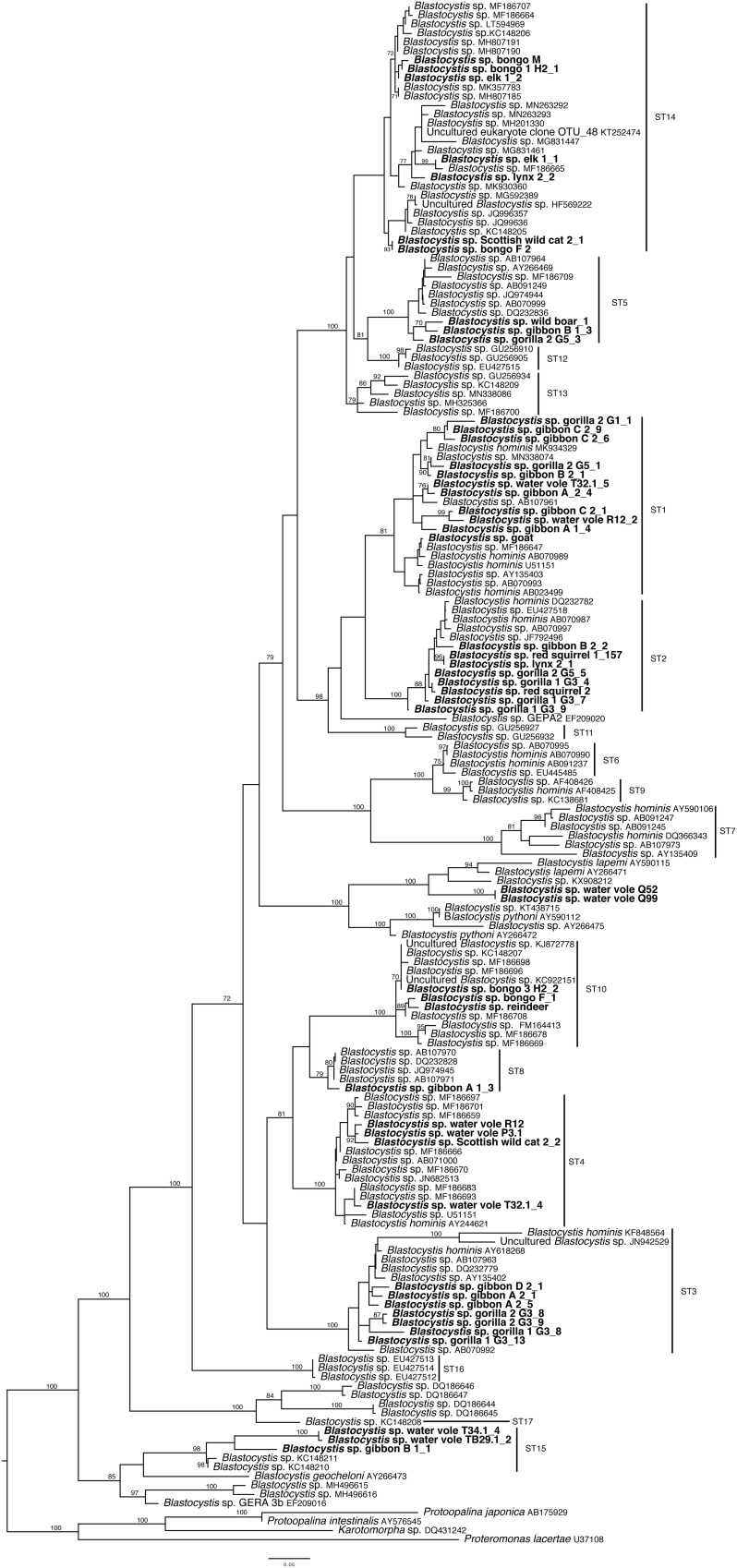
Maximum likelihood phylogenetic tree inferred from 171 sequences and 1326 sites using RAxML v. 8. New sequences are in bold lettering. Numerical values indicate bootstrap support values and only those of over 70 are shown.

All *Blastocystis* sequences grouped together with maximum support (100BS) (Figure 1). Newly acquired sequences belong to ST1, ST2, ST3, ST4, ST5, ST8, ST10, ST14, ST15, and the *B. lapemi* clade. In agreement with previous studies, ST15, ST16 and ST17 along with sequences originating from ectotherms placed in the most basal positions (Alfellani et al., 2013; Yowang et al., 2018). Subtypes 3, 4, 8, and 10 grouped together, while subtypes 7, 9 and 6 formed a clade. Two of the water vole sequences grouped within the clade formed by *B. lapemi* and *B. pythoni*. Subtypes 1, 2 and 11 grouped together and sister to the clade formed by subtypes 5, 12, 13, and 14.

## Discussion

Animals from 38 species from two animal parks in the United Kingdom were sampled over a period of 3 years. Eighty-two samples from 47% of all animal species were sequence positive for *Blastocystis*. Of those 82, (21/82) 26% were found to harbor more than one ST, while 53% also harbored other protists. *Blastocystis* was present in animals from both parks. As expected, ST4 was dominant in rodents, whereas ST10 and ST14 dominated in artiodactyls. In primates, ST1 and ST2 were dominant. We reported *Blastocystis* presence in the Lynx and the Scottish wild cat for the first time. Both of these animals are carnivorous. Our study confirms previous findings on reduced presence and often absence of *Blastocystis* in carnivores and high prevalence in artiodactyls (Alfellani et al., 2013; Cian et al., 2017, Zhao et al., 2017). It is well known that dietary, behavioral and environmental factors shape bacterial communities, though this has yet to be shown for microbial eukaryotes. In that vein, a possible explanation for the above observation could be that captive carnivores consume a diet consisting of almost exclusively refrigerated meat, which is devoid of other eukaryotes. This considerably reduces contamination. Nonetheless, a recent study on free-living carnivorous animals confirmed presence of *Blastocystis* in only 1.6% of hosts (Calero-Bernal et al., 2019), suggesting that additional factors might account for the low prevalence. Artiodactyls are herbivorous animals that consume exclusively fiber, while carnivores consume only animal protein. Thus the two also differ considerably in the overall structure and physiology of their respective gastrointestinal tracts. Both diet and physiology likely contribute to microbiota composition, and as a result, the microbial communities of artiodactyls and carnivores differ considerably (Sanders et al., 2015; Nishida and Ochman, 2018). In general, herbivores, to which artiodactyls belong, harbor high microbial diversity, while carnivores encompass the least diverse microbial communities amongst mammals (Nishida and Ochman, 2018). High microbial diversity and specific microbial profiles are linked to presence of *Blastocystis* in human studies though a causative link has yet to be established (Andersen et al., 2015; Audebert et al., 2016; Iebba et al., 2016; O’Brien Andersen et al., 2016; Beghini et al., 2017; Forsell et al., 2017; Nieves-Ramirez et al., 2018; Tito et al., 2019). A similar result has also been obtained from a study focusing on wild chimpanzees (Renelies-Hamilton et al., 2019). Given the high prevalence of *Blastocystis* in artiodactyls it would be interesting to explore whether such specific profiles exist in these animals as well.

As in our previous study (Betts et al., 2018), we identified multiple subtypes of *Blastocystis* in the same host. In addition to the elk, pygmy goat, red deer and water vole hosts bearing multiple subtypes, we add the Scottish wildcat (ST4 and ST14), bongo (ST10, ST14), and lynx (ST2, ST14). Previous reports also noticed presence of multiple STs in animals (Fayer et al., 2012; Badparva et al., 2015; AbuOdeh et al., 2016). Cian et al., documented several instances of mixed colonization of subtypes (11%), especially in primates and artiodactyls (Cian et al., 2017), while Wang et al., reported mixed colonization in 58% of a pig population (Wang et al., 2014). Collectively these data strengthen previously raised hypotheses that occurrence of multiple subtypes in animals is not unusual, but rather common (Fayer et al., 2012; Betts et al., 2018). Thus, a logical extension of this study would be to disentangle whether co-occurring subtypes occupy distinct functional niches in the complex gut ecosystem, a direction that has also been suggested by Beghini et al. (2017).

Co-occurrence of *Blastocystis* with *Entamoeba*, *Giardia*, *Cryptosporidium* and *Eimeria* in multiple animal species across the two parks was also examined. Most previous studies have either looked for multiple parasites from single animal species or have targeted one microbial eukaryote in various hosts (Fayer et al., 2012; Parsons et al., 2015; Enriquez et al., 2016, 2019; Jacob et al., 2016). Herein, *Blastocystis* did not co-occur with other protists in any of the carnivores, even though we did observe co-occurrence of *Cryptosporidium* and *Eimeria* in gray and Iberian wolves. The case of artiodactyls is particularly notable. Eight out of ten artiodactyls that were *Blastocystis* positives co-occurred with an *Entamoeba* species. Out of these, three co-occurred with *Blastocystis*, *Entamoeba* and *Eimeria* (European bison, reindeer, bongo), while one animal had *Blastocystis*, *Entamoeba* and *Cryptosporidium* (red river hog). Significantly, none of the animals exhibited diarrheal episodes or other obvious gastrointestinal symptoms as confirmed by zookeepers and licensed veterinarians. Typically, microbial eukaryotes in animals are identified and reported upon onset of gastrointestinal symptoms. Herein, we sampled and detected gut protists before presentation of symptoms, though the possibility that some of the animals might have had symptoms before they were brought into the parks cannot be excluded. Asymptomatic carriage of a single or multiple protists in animals is not uncommon and the concern of zoonotic transmission has often been articulated (Fayer et al., 2012; Cian et al., 2017; Desoubeaux et al., 2018; Udonsom et al., 2018; Wang et al., 2018a; Enriquez et al., 2019). In case of zoonosis, detecting the reservoir is difficult as there is no reason to check the original host for presence of pathogens. The level and type of interaction among *Blastocystis*, other microbial eukaryotes (including fungi) and the rest of the host microbiome is unclear. Future animal studies should focus on exploring the eukaryotic component of the gut microbiome rather than targeting individual microbial species, in order to shed light on the role of eukaryome as a whole in the gut ecosystem. Combination of *in vitro* and *in vivo* targeted metagenomics and metabolomics approaches along with network analysis will greatly increase our understanding of these issues.

The case of *Blastocystis* is of interest. In the past, co-occurrence of *Blastocystis* with pathogens in stool samples of humans with gastrointestinal symptoms was likely one of the reasons for its controversial pathogenicity. Since adaptation of the subtyping system, the argument has been framed around specific subtypes or strains being pathogenic. Nonetheless, in a rather anthropocentric approach, assessment of the pathogenic potential of *Blastocystis* has focused primarily on humans and the “human” subtypes ST1 to ST9, while non-human metazoans and the rest of the subtypes have been largely overlooked. Moreover, the health status of animal subjects in many studies is not reported. When animals happen to have diarrhea the subtype present in these animals is often not mentioned, rather percent overall occurrence of individual subtypes is emphasized. Consequently, *Blastocystis* pathogenicity in animals is not well understood. It would be interesting to see whether any of the animals sampled herein will present any symptoms in the future. To that end, we have communicated with the zoo stuff to inform us in case symptoms develop in any of these animals.

To determine to which subtype the new sequences belonged, phylogenetic analysis was performed. Two of the newly generated sequences, both of which come from water voles, did not group with any of the known subtypes, but as sister to *Blastocystis lapemi*. There are two sequences designated as *B. lapemi* in the database, both of which originated from sea snakes (Yoshikawa et al., 2004; Noel et al., 2005). A third sequence that also groups within the clade and is genetically distinct comes from a monitor lizard. Therefore, either *B. lapemi* is not limited to sea snakes or all these sequences represent different species. In the absence of a culture and a full SSU rRNA sequence we designate those three sequences as *Blastocystis* sp. Four sequences – one coming from gibbon and three from water voles – group with ST15. Water vole is a newly reported host for ST15. Previously, Betts et al. (2018) had reported a potentially novel subtype, but had refrained from establishing it as such since the whole sequence was not available. Since then, several studies focusing on animals have contributed significantly toward populating previously isolate-sparse subtypes. As a result, the phylogenetic landscape of *Blastocystis* is changing. Expanded taxon sampling including several additional ST14 isolates from the database and from the current study has shown that ST14 is now divided into three distinct subclades, with new isolates populating all three. The previously suspected novel sequence (Betts et al., 2018) groups in one of the three. Thus, either ST14 has high intra-subtype divergence or it must be separated to at least two maybe even three subtypes. Nonetheless several subtypes harbor a high degree of genetic diversity except for ST4, which is the least genetically diverse (Stensvold and Clark, 2016b; Beghini et al., 2017). Given the variable degree of intra-subtype diversity, caution should be taken when establishing new subtypes. Genetic diversity within subtypes should be properly assessed. Commonly, closely related sequences from specific subtypes are included in the analysis, while more divergent representatives are not, leading to establishment of erroneous STs. Finally, the whole SSU rRNA region should be sequenced and phylogenies should include the breadth of *Blastocystis* diversity. Consistent approaches to subtyping *Blastocystis* will further elucidate the variety of subtypes that exist and their associations with specific hosts (El Safadi et al., 2016; Betts et al., 2018; Robertson et al., 2019).

In the current study, we employed cloning and demonstrated the presence of multiple subtypes within a single host and also presence of multiple eukaryotes within a host. We would like to emphasize that DNA was mainly extracted directly from fresh fecal samples without culturing in Jones media. Even though we still cannot guarantee that all subtypes present in the stool samples were amplified, selective pressures and constraints that culturing imposes were circumvented. In working with fecal samples other issues came to light. One of them is primer specificity. Eukaryotic microbe primers amplify the microbe of interest provided it is there. Our screening showed that all pairs of specific primers and most especially those of *Blastocystis* and *Entamoeba* also amplified several other eukaryotes. For example, approximately ∼40% of the sequenced clones did not correspond to *Blastocystis* specific sequences. Development of new *Blastocystis-*specific primers that will amplify a large fragment of the SSU rRNA gene are urgently needed, since this will reduce the costs of cloning and sequencing.

## Conclusion

Herein we have identified asymptomatic carriage of multiple microbial eukaryotes in a number of animal species. This is defined as presence of multiple *Blastocystis* subtypes in single hosts and in many cases these co-occur with up to three other microbial eukaryotes. Given the higher prevalence of overlap of microbial eukaryotes in animals and especially in artiodactyls, the latter might provide a model not only for studying the spectrum of parasitism (Rueckert et al., 2019), but also the associated microbial communities and how those relate with the different parts of this spectrum.

## Data Availability Statement

The raw data supporting the conclusions of this article will be made available by the authors, without undue reservation, to any qualified researcher.

## Author Contributions

EB carried out the collections, culturing, collected and analyzed all the data, and wrote a first draft of the manuscript. AT and EG directed research, planned experiments, analyzed data, and wrote the manuscript.

## Conflict of Interest

The authors declare that the research was conducted in the absence of any commercial or financial relationships that could be construed as a potential conflict of interest.

## Footnotes

1 http://entamoeba.lshtm.ac.uk/xenic.htm

2 http://www.phylo.org/
