# Supplementary material for: Exploring Micro-Eukaryotic Diversity in the Gut: Co-occurrence of Blastocystis Subtypes and Other Protists in Zoo Animals
Source: Front Microbiol. 2020 Feb 25;11:288. doi: 10.3389/fmicb.2020.00288 (PMC7052370; doi:10.3389/fmicb.2020.00288)
Supplement: Supplementary file 1 [file Table_1.DOCX]

| Host  **Supplementary Table 1:** Animals sampled in this study | Scientific Name | Location | Collection Date(s) mm/yy | Total number of faecal samples |
| --- | --- | --- | --- | --- |
| Carnivora (T=50) | | | |  |
| Badger | *Meles meles* | Wildwood | 10/16 | 2 |
| European Brown Bear | *Ursus arctos arctos* | Wildwood | 10/16 - 05/18 | 4 |
| Lynx | *Lynx lynx* | Wildwood | 10/16 - 05/18 | 5 |
| Otter | *Lutra lutra* | Wildwood | 10/16 | 7 |
| Pine Marten | *Martes martes* | Wildwood | 10/16 | 2 |
| Polecat | *Mustela putarius* | Wildwood | 10/16 | 1 |
| Red Fox | *Vulpes vulpes* | Wildwood | 10/16 | 3 |
| Arctic Fox | *Vulpes lagopus* | Wildwood | 05/18 | 2 |
| Scottish Wild Cat | *Felis silvestris* | Wildwood | 10/16 – 05/18 | 13 |
| Stoat | *Mustela ermine* | Wildwood | 10/16 | 3 |
| Grey Wolf | *Canis lupus* | Howletts | 11/17 – 02/19 | 3 |
| Grey Wolf | *Canis lupus* | Wildwood | 08/18 | 2 |
| Iberian Wolf | *Canis lupus signatus* | Howletts | 11/17 – 02/19 | 3 |
| Anseriformes (T=2) | | | | |
| Barnacle Goose | *Branta leucopsis* | Wildwood | 10/16 | 1 |
| Pink Footed Goose | *Anser brachyrhynchus* | Wildwood | 10/16 | 1 |
| Artiodactyla (T=36) | | | | |
| Muntjac | *Muntiacus reevesi* | Wildwood | 10/16 | 1 |
| European Bison | *Bison bonasus* | Wildwood | 07/16 – 02/19 | 5 |
| European Bison | *Bison bonasus* | Howletts | 11/17 – 02/19 | 4 |
| Eurasian Elk | *Alces alces* | Wildwood | 10/16 | 3 |
| Pygmy Goat | *Capra aegagrus hircus* | Wildwood | 10/16 | 2 |
| Red Deer | *Cervus elaphus* | Wildwood | 07/16 – 02/19 | 3 |
| Reindeer | *Rangifer tarandus* | Wildwood | 10/16 | 1 |
| Soay Sheep | *Ovis aries* | Wildwood | 10/16 | 1 |
| Wild Boar | *Sus scrofa* | Wildwood | 10/16 | 4 |
| Red River Hog | *Potamochoerus porcus* | Howletts | 11/17 – 02/19 | 6 |
| Bongo | *Tragelaphus eurycerus* | Howletts | 11/17 – 02/19 | 6 |
| Squamata (T=1) | | | | |
| Four-lined Snake | *Elaphe quatuorlineata* | Wildwood | 10/16 | 1 |
| Eulopotyphla (T=7) |  |  |  |  |
| Hedgehog | *Erinaceus quatuorlineata* | Wildwood | 10/16 | 1 |
| Water Shrew | *Neomys fodiens* | Wildwood | 07/16 | 6 |
| Passeriformes (T=4) | | | | |
| Raven | *Corvus corax* | Wildwood | 01/17 – 05/18 | 3 |
| Red Billed Chough | *Pyrrhocorax pyrrhocorax* | Wildwood | 10/16 | 1 |
| Rodentia (T=81) | | | | |
| Black Rat | *Rattus rattus* | Wildwood | 05/18 | 1 |
| Brown Rat | *Rattus norvegicus* | Wildwood | 05/18 | 1 |
| Red Squirrel | *Sciurus vulgaris* | Wildwood | 07/16 – 02/19 | 5 |
| Water Vole | *Arvicola amphibious* | Wildwood | 07/16 – 03/19 | 22 |
| Water Vole | *Arvicola amphibious* | Tilbury | 09/16 – 04/17 | 17 |
| Water Vole | *Arvicola amphibious* | Bulphan | 10/16 – 03/17 | 35 |
| Diprotodontia (T=5) | | | | |
| Wallaby | *Macropus rufogriseus* | Wildwood | 07/16 – 05/18 | 5 |
| Primate (T=43) | | | | |
| Western Lowland Gorilla | *Gorilla gorilla gorilla* | Howletts | 11/17 – 02/19 | 25 |
| Javan Gibbon | *Hylobates moloch* | Howletts | 11/17 – 02/19 | 13 |
| Pied Tamarin | *Saguinus bicolor* | Howletts | 11/17 – 02/19 | 5 |
| [Perissodactyla](https://en.wikipedia.org/wiki/Odd-toed_ungulate) (T=2) | | | | |
| Black Rhinoceros | *Diceros bicornis* | Howletts | 11/17 – 02/19 | 2 |
