# Supplementary material for: Exploring Micro-Eukaryotic Diversity in the Gut: Co-occurrence of Blastocystis Subtypes and Other Protists in Zoo Animals
Source: Front Microbiol. 2020 Feb 25;11:288. doi: 10.3389/fmicb.2020.00288 (PMC7052370; doi:10.3389/fmicb.2020.00288)
Supplement: Supplementary file 2 [file Table_2.DOCX]

**Supplementary Table 2:** PCR primers used in this study

| Primer Pair | Target Loci | Organism of Interest | Primers | | |
| --- | --- | --- | --- | --- | --- |
|  |  |  | Band Size /bp | Sequence 5’-3’ | Reference |
| RD3 | SSUrRNA | *Blastocystis ST* | - | GGGATCCTGATCCTTCCGCAGGTTCACCTAC | Clark, 1997 |
| RD5 | SSUrRNA | *Blastocystis ST* | - | GGAAGCTTATCTGGTTGATCCTGCCAGTA |  |
| RD5F | SSUrRNA | *Blastocystis ST* | 650 | ATCTGGTTGATCCTGCCAGT | Scicluna, 2006 |
| BhRDr | SSUrRNA | *Blastocystis ST* |  | GAGCTTTTTAACTGCAACAACG |  |
| CRY-F1 | SSUrRNA | *Cryptosporidium sp* | - | GATTAAGCCATGCATGTCTAAG |  |
| CRY-R1 | SSUrRNA | *Cryptosporidium sp* |  | TTCCATGCTGGAGTATTCAAG |  |
| CRY-F2 | SSUrRNA | *Cryptosporidium sp* | 638 | CAGTTATAGTTTACTTGATAATC |  |
| CRY-R2 | SSUrRNA | *Cryptosporidium sp* |  | CCTGCTTTAAGCACTCTAATTTTC |  |
| RH11 | SSUrRNA | *Giardia sp* | 292 | CATCCGGTCGATCCTGCC | Hopkins, 1997 |
| RH4 | SSUrRNA | *Giardia sp* |  | AGTCGAAC CCTGATTCTCCGCCCAGG |  |
| 542 | SSUrRNA | *Entamoeba sp* | 450 | GTTGATCCTGCCAAGTATTATATGCT | Clark, 2006 |
| 543 | SSUrRNA | *Entamoeba sp* |  | GACTATTGGAGCTGGAATTACCG |  |
| EIF1 | SSUrRNA | *Eimeria sp* | ≈ 1400 | GCTTGTCTCAAAGATTAAGCC | Zhao, 2001 rDNA  Power, 2009 rDNA  Yang, 2012 rRNA |
| EIR3 | SSUrRNA | *Eimeria sp* |  | ATGCATACTCAAAAGATTACC |  |
| EIF3 | SSUrRNA | *Eimeria sp* |  | CTATGGCTAATACATGCGCAATC |  |
