# Supplementary figures and images for: Exploring Micro-Eukaryotic Diversity in the Gut: Co-occurrence of Blastocystis Subtypes and Other Protists in Zoo Animals
Source: Front Microbiol. 2020 Feb 25;11:288. doi: 10.3389/fmicb.2020.00288 (PMC7052370; doi:10.3389/fmicb.2020.00288)

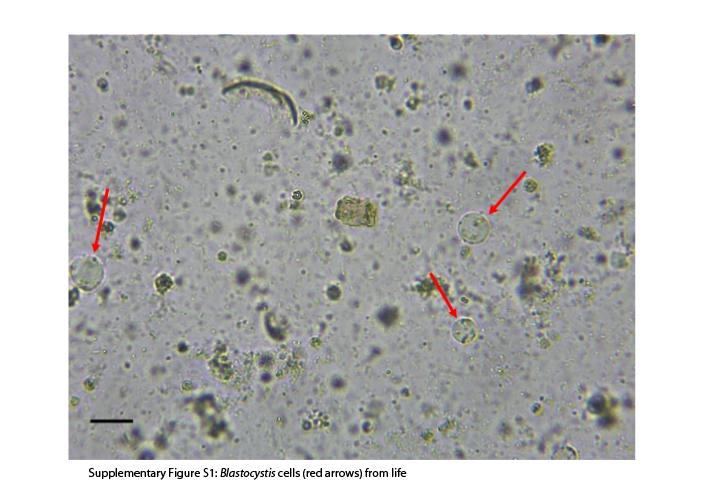

Supplement: Supplementary file 3 [file Image_1.tif]
